# *The Organon* and Materia Medica Club of the Bay Cities of California

**Published:** 1898-05

**Authors:** Guy E. Manning

**Affiliations:** Secretary


### *THE ORGANON* AND MATERIA MEDICA CLUB OF THE BAY CITIES OF CALIFORNIA.

Meeting called to order September 17, 1897, at 8.20 P. M. by the President, Dr. Selfridge.

Present—Drs. J. M. Selfridge, G. H. and E. F. Martin, Augur, J. W. and F. N. Ward, Holmgren, and Manning.

The minutes of the previous meeting read and accepted.

Dr. Manning read a paper upon "The First Caution of Hahnemann," which dealt with the words of the leader, in which he (Hahnemann) cautions his followers against interfering with the good results of a drug by replacing it with some other remedy to meet the fleeting symptoms:

"If you have chosen the remedy carefully and with study you are not to be alarmed by other symptoms. On closer questioning it may be determined that these symptoms were previously felt, and it may be considered that the remedy in its beneficial effects has set up these symptoms; or if they have never been present, and now continue to increase in severity, the supposition then is that a wrong remedy has been selected. Its action then is to be antidoted and a new remedy selected. Or there may be an aggravation of the prevailing symptoms which, if they soon decrease or disappear, is to be considered as due to a homœopathic aggravation, and no change should be made; but if increasing in severity then it is to be considered as a drug effect, and the dose is too large. The medicine is then to be stopped, and when necessary to continue is to be given in a higher attenuation."

DISCUSSION.

Dr. Manning—These are the thoughts of Hahnemann and to some extent his words, and while I believe in his theories, and do not attempt to criticise them, yet I find it hard to hold myself to them, nor have I done so. So much depends upon one's early training.

While at college we received practically no teaching or insight into *The Organon*. What, then, can you expect? Then in the giving of high attenuations and the infrequent dosing one should feel confident of his materia medica. I think one should report here his difficulties and failures as well as all successful cases. Now, I also have trouble in the selection of my remedy. If I make special endeavors, using checking list and repertory, it seems to me that in nine cases out of ten I arrive at Sulphur. Now I am aware that Sulphur is a great remedy, and so many of our chronic diseases are due to psora, and Sulphur is our great antipsoric, it may be all right, but I'd like a change once in a while and I would feel better. Now the question arises, do I fail to take my case right, or is proper significance given to the weighty symptoms?

Dr. Selfridge—The doctor has probably made his mistakes through improper education at college. He was not started right. The greatest thing is to examine the patient properly, and Hahnemann's method cannot be improved upon. Then comes the choice of remedy. We gain nothing by alternation. Choose the single remedy. Then if you fail you know why. I am guided in the repetition of the dose by the rapidity of the disease. In pleurisy, pneumonia, etc.—dangerous diseases, exhausting the system in a few days—it is necessary to repeat. In chronic diseases all are apt to give too often. Take scrofula, tumors, and such long lasting diseases; my plan is not to give oftener than thirty days. A very important part is to take the case properly. We get two, three or four symptoms and think that's enough. Obtain particularly the mental symptoms.

Dr. Martin—I do not think there are many real true homœopathic prescriptions given where we have to prescribe in the office or on the spur of the moment, because that is a very difficult thing to do, and the reason of many of our cures is the fact that the remedy comes near many of the symptoms. It is not possible to get every symptom of every case. If we get the main symptoms then we can get near the remedy. Then if we go farther we are apt to get a keynote that will at once give us the correct remedy. In chronic cases there is the trouble of giving doses too often. I believe that an intense disease requires more frequent medicine. My plan is to give frequently until I see some improvement or change; then lessen frequency.

Dr. Augur—I was started in the right way, and try to give according to Hahnemann's doctrines.

Dr. Manning—Would like to ask Dr. Selfridge if he always takes the family history, and if so how much he is governed by it in prescribing?

Dr. Selfridge—I always give a nosode at some time during treatment if family history shows need.

Dr. Martin—Would not Tuberculin be indicated in every case of consumption; Syphilinum in every case of syphilis, and so on?

Dr. Selfridge—I think I would choose them as my first remedies, unless other remedies were especially indicated.

Dr. Martin—Because Tuberculin is prepared from tuberculous product is where isopathy falls through, and why we can't cure every case with Tuberculinum, Syphilinum, etc. Here is the point: Give half a dozen people Tuberculin and different symptoms will develop, due to different constitutions.

Dr. F. N. Ward—In the homœopathic production what is taken, tubercles, bacilli, ptomaines, or what?

Dr. Selfridge—In Swan's Tuberculinum-album I infer that it is taken from the crude tubercle of the diseased lung, but it may be from sputum.

Dr. F. N. Ward—Modern investigation shows that symptoms vary according to whether the Tuberculin is derived from tubercle bacilli or ptomaines.

Discussion was then closed, and Dr. Manning appointed essayist for the next meeting.

Adjourned on motion.

GUY E. MANNING,
*Secretary.*

